# Elevated Transaminases Revealing Celiac Disease in a Patient With Normal Gastrointestinal Symptoms in a Tertiary Healthcare Setting: A Case Report

**DOI:** 10.7759/cureus.96240

**Published:** 2025-11-06

**Authors:** Farya Mughal, Ahmad Munib, Amanullah Khan

**Affiliations:** 1 Intensive Care Unit, The Clementine Churchill Hospital, London, GBR; 2 Department of Internal Medicine, Shaikh Zayed Hospital, Lahore, PAK; 3 Medical and Health Research, Punjab University, Lahore, PAK; 4 Medical Research, University of Health Sciences, Lahore, PAK

**Keywords:** asymptomatic diseases, celiac disease, gluten-free diet, hypertransaminasemia, liver function tests

## Abstract

Celiac disease (CD) is a lasting immune disease, most commonly with gastrointestinal symptoms; nonetheless, atypical symptoms of non-digestive tracts have now increasingly become common. We report the case of a 28-year-old woman with a history of constantly increased transaminases and no gastrointestinal symptoms. An extensive hepatic etiology testing was of no note. CD was diagnosed through the presence of positive anti-tissue transglutaminase IgA and duodenal biopsy. There was a great improvement, including liver enzyme normalization, in three months of a gluten-free diet. This case showed the necessity to consider CD as part of the diagnostic process of unexplained hypertransaminasemia despite the lack of gastrointestinal symptoms. Preventive measures of hepatic progression to widespread involvement and an increase in long-term prognosis can be achieved through dietary intervention and early diagnosis.

## Introduction

Celiac disease (CD) is a chronic immune-mediated disorder that is characterized by the resultant deviant immune response to gluten in genetically susceptible individuals. It is normally an intestinal ailment, but there is an emerging fact that it may have numerous extra-intestinal manifestations [1]. The hepatic involvement is one of the non-classical manifestations, most of the time appearing as serum transaminase elevation, although it is not accompanied by gastrointestinal distress. The most frequent causes that are taken into consideration in clinical practice in case of a persistent elevation of liver enzymes include viral hepatitis, autoimmune hepatitis, metabolic disorders, and drug-induced hepatotoxicity [2]. However, when these usual causes are eliminated, the etiological cause is normally not known. Although CD has structured systemic manifestations, it remains undetected in the development of the differential diagnosis of cryptogenic hypertransaminasemia, mostly in patients who do not have gastrointestinal symptoms or nutritional deficiencies [3].

It is not known in detail how the pathophysiological processes linking CD and liver dysfunction work; potential mechanisms are an augmented intestinal permeability, immunologically mediated liver damage, and circulating autoantibodies [4]. Interestingly, the development of such a hepatic dysfunction can be corrected with the introduction of a strict gluten-free diet (GFD), which means that liver enzyme levels may be restored to normal and the disease will not progress due to a non-pharmacologic form of treatment [5]. In this case report, an adult woman was referred to the center with the only finding of hypertransaminasemia, without any gastrointestinal or systemic symptoms. Serological and histopathological diagnosis of CD, along with the restoration of normal liver enzyme after education on the diet, demonstrates the necessity of improved clinical awareness of CD in patients with an abnormal liver enzyme etiology.

## Case presentation

A 28-year-old female patient from Punjab University-affiliated tertiary healthcare settings in Lahore was referred to the hepatology clinic after persistently elevated liver transaminases were detected during a routine employment screening. She denied abdominal pain, diarrhea, bloating, weight loss, fatigue, or any other gastrointestinal complaints. Her appetite and dietary preferences were normal for her region. She denied alcohol use, herbal supplements, or hepatotoxic medications. The patient’s family history was negative for autoimmune or gastrointestinal diseases. Physical examination revealed that she was hemodynamically stable and well nourished. There was no evidence of chronic liver disease such as jaundice, palmar erythema, and hepatosplenomegaly. Her body mass index was 22.4 kg/m², within the normal range. Laboratory tests showed elevated aminotransferase levels at baseline while synthetic liver functions, complete blood counts, and cholestatic enzymes were normal.

A hepatic workup was carried out thoroughly. Viral hepatitis serologies (HAV IgM, HBsAg, anti-HCV, and HEV IgM) were negative. The thyroid profile was normal, as shown in Table 1. Hepatobiliary ultrasound imaging showed a normal hepatobiliary size and echotexture without any steatosis, localized lesion, or biliary duct dilatation. Portal and hepatic veins were patent, and the size of the spleen was normal.

**Table 1:** Hepatic workup and imaging findings prior to celiac disease diagnosis. Screening of autoimmune hepatitis with antinuclear antibody (ANA), anti-smooth muscle antibody (ASMA), and anti-liver kidney microsomal antibody type 1 (anti-LKM-1) was negative. Markers of primary biliary cholangitis including anti-mitochondrial antibody (AMA) were not positive. The level of iron showed normal ferritin (86 µg/L) and saturation of transferrin (28%), eliminating hemochromatosis. Ceruloplasmin was also normal (31 mg/dL) which ruled out Wilson disease. NA: Not applicable; TSH: thyroid-stimulating hormone

Test Category	Specific Test/Parameter	Result	Reference Range	Interpretation
Viral hepatitis screening	HAV IgM, HBsAg, Anti-HCV Ab, HEV IgM	Negative	NA	No viral infection detected
Autoimmune hepatitis (AIH) panel	ANA (titer < 1:40), ASMA < 1:40, Anti-LKM-1 Ab Negative	Negative	ANA < 1:40, ASMA < 1:40, Anti-LKM-1 Ab Negative	No evidence of autoimmune hepatitis
Primary biliary cholangitis (PBC)	Anti-mitochondrial antibody (AMA M2)	Negative	NA	PBC excluded
Hemochromatosis screen	Serum ferritin 86 µg/L; Transferrin saturation 28 %	Ferritin within normal limits; Transferrin saturation normal	Ferritin: 30–400 µg/L (men), 15–150 µg/L (women); Transferrin saturation: 20–45 %	No iron overload
Wilson’s disease screen	Serum ceruloplasmin 31 mg/dL	Normal	20–35 mg/dL	Wilson’s disease excluded
Thyroid Function	TSH 2.1 mIU/L	Normal	0.4–4.0 mIU/L	No thyroid dysfunction
Hepatobiliary Ultrasound	Normal liver size (13.2 cm), homogeneous echotexture; no focal lesion, no biliary dilatation; portal and hepatic veins patent; spleen normal (10.5 cm)	Normal study	—	No structural or vascular abnormality

As no hepatic etiology could be identified and transaminases remained persistently elevated, screening for CD was performed. Anti-tissue transglutaminase (anti-tTG) IgA levels were remarkably elevated (89 U/mL) and anti-endomysial IgA levels were strongly positive (titer 1:160). Upper endoscopy revealed scalloping of the duodenal folds. The second part of duodenal biopsies demonstrated crypt hyperplasia, villous blunting, and increased intraepithelial lymphocytes (> 40 per 100 enterocytes) consistent with Marsh IIIa. The patient was started on a strict GFD under the supervision of a clinical dietitian. Clinical stability, complete normalization of hepatic enzymes, and serologic remission were observed at six months of follow-up. Table 2 summarizes the clinical and laboratory parameters at baseline and after six months of treatment.

**Table 2:** Laboratory and serologic parameters at baseline and after six months on a gluten-free diet AST: Aspartate Aminotransferase; ALT: Alanine Aminotransferase; ALP: Alkaline Phosphatase; INR: International Normalized Ratio; tTG IgA: Tissue Transglutaminase Immunoglobulin A; EMA IgA: Endomysial Antibody Immunoglobulin A

Parameter	At Baseline	After Six Months	Reference Range
Platelet count (×10⁹/L)	216	235	150–450
Hemoglobin (g/dL)	12.8	13.7	12–16
White blood cell count	6.2	6.5	4.0–10.0 ×10⁹/L
Mean corpuscular volume (fL)	85	86	80–98
AST (U/L)	108	34	5–40
ALT (U/L)	122	29	7–56
ALP (U/L)	93	87	44–147
Total bilirubin (mg/dL)	0.8	0.7	0.2–1.2
Albumin (g/dL)	3.9	4.3	3.5–5.0
INR	1.0	1.0	0.8–1.2
Anti-tTG IgA (U/mL)	89	<10	<20
Anti-endomysial IgA	Positive (1:160)	Negative	Negative

**Figure 1:**
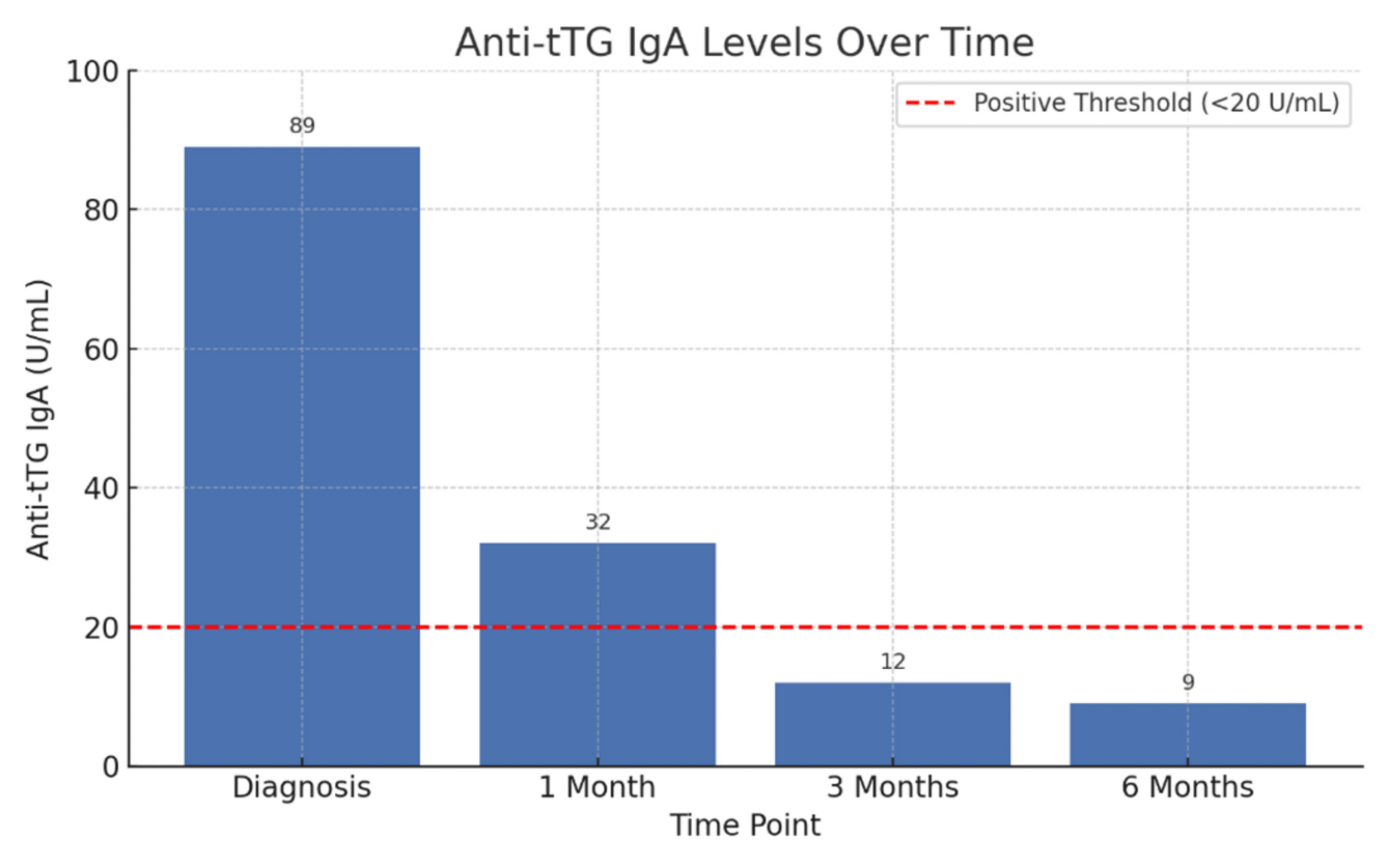
Bar chart showing anti-tissue transglutaminase immunoglobulin A levels at diagnosis and during follow-up.

After a GFD was adopted, liver transaminases returned to normal, and celiac-specific antibodies declined, reflecting both biochemical and immunologic remission. The titers of the celiac-specific antibodies decreased gradually with adherence to a GFD. Figure 1 illustrates the changes in anti-tTG IgA levels during follow-up.

**Figure 2:**
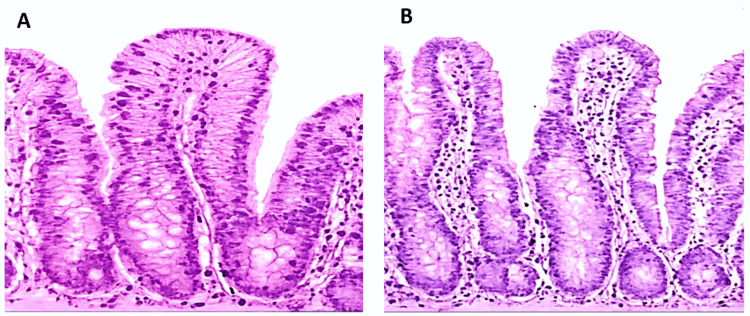
Duodenal biopsy; pre and post gluten-free diet and mucosal healing. Part A (left) shows villous blunting, hyperplastic crypts and increased intraepithelial lymphocytes (Marsh IIIa) in the first duodenal biopsy. Part B (right) represents the follow-up duodenal biopsy after 12 months, showing normal villous architecture with minimal intraepithelial lymphocytosis.

At baseline, the anti-tTG IgA levels were 89 U/mL, while within the third month, they had dropped below the positive threshold (<20 U/mL), indicating immunologic remission with strict adherence to the GFD. A repeat duodenal biopsy was performed after 12 months to determine mucosal recovery, which showed histologic remission, such as normalization of villous architecture and decreased intraepithelial lymphocytes (<25 per 100 cells). Figure 2 illustrates histologic changes from baseline to one year.

The first biopsy showed a case of villous blunting, hyperplasia of the crypt, and an augmentation of intraepithelial lymphocytes, which are in line with Marsh IIIa CD. A follow-up biopsy was taken after 12 months of GFD and this showed full mucosal recovery, which proved the ability to manage the diet and achieve remission. Histological reports are summarized in Table 3.

**Table 3:** Histopathological findings of duodenal biopsies. IEL: Intraepithelial Lymphocytes

Biopsy Site	Findings	Interpretation
Duodenum (initial)	Villous blunting, crypt hyperplasia, IEL >40/100 enterocytes	Marsh IIIa – Active celiac disease
Duodenum (12 months)	Normal villous architecture, IEL <25/100 enterocytes	Mucosal recovery with gluten-free diet

The diagnosis of CD was confirmed histologically at baseline and showed mucosal healing after one year of adherence to the diet.

## Discussion

This case reflects an abnormal demonstration of CD in a 28-year-old woman with persistent elevation of liver transaminases without coincident gastrointestinal symptoms. Although the patient did not experience any digestive symptoms, both biochemical evidence and histologic evidence of active CD were present. Serologic remission and normalization of liver enzymes after initiation of a GFD provide strong evidence in favor of a causal relationship between subclinical CD and liver involvement.

Isolated hypertransaminasemia in celiac patients without abdominal discomfort is recognized, but usually underdiagnosed [6]. A number of cases have been reported where elevated asymptomatic transaminases were the only manifestation of CD, particularly in adults undergoing regular screening or being evaluated for mild abnormalities of liver tests [7]. In such cases, the derangement of the liver enzymes is non-specific and mild to moderate. Notably, the appearance of liver abnormalities that are responsive to strict gluten withdrawal, as demonstrated in this patient, is a characteristic feature of celiac hepatopathy [8]. It is believed that the underlying process is associated with immune-mediated inflammation, increased intestinal permeability, and liver exposure to antigens of gluten [9].

The patient did not have any other identifiable cause of elevated liver enzymes after a comprehensive search, including a negative workup for viral, autoimmune, and metabolic causes. Further supportive evidence for the diagnosis and therapeutic response includes the rapid decline in anti-tTG IgA levels, seroconversion of anti-endomysial antibodies, and histologic mucosal healing after a GFD [10]. These findings further strengthen the role of celiac serology within the diagnostic algorithm of cryptogenic transaminasemia, even in the absence of gastrointestinal symptoms. Clinically, the case highlights the importance of considering CD as an uncommon cause of hypertransaminasemia within the differential diagnosis when typical hepatic pathologies have been excluded. The diagnostic rate of celiac serology in such scenarios is clinically relevant, and early diagnosis allows reversal of liver abnormalities with dietary intervention alone [11].

However, the limitations can be added as no liver biopsy was carried out, which would have given clear histological proof of hepatic involvement. Here, liver biopsy was not performed due to the fact that all the serologic and imaging studies were normal, and the patient was showing a full biochemical and serologic recovery in three months of a GFD. This is a typical reaction of celiac-related liver damage also known as celiac hepatitis, which usually vanishes following withdrawal of gluten and without the necessity of invasive hepatic biopsy. Nevertheless, restoring liver biochemistry and duodenal architecture to a normal state in the context of a GFD may have strong circumstantial evidence of gluten-mediated liver damage. Further studies are needed to better characterize the spectrum of liver abnormalities in subclinical cases of CD and to establish long-term liver outcomes in such patients.

## Conclusions

This case reflected the importance of considering CD in the differential diagnosis of isolated elevation of transaminases, despite the absence of gastrointestinal symptoms. Complete mucosal and biochemical healing can occur when subclinical CD is identified in patients with serologic and histologic evidence of disease, followed by strict adherence to a GFD.

When standard hepatic protocols are unrewarding, clinicians should maintain a high index of suspicion for atypical or silent presentations of CD. It is vital to note the systemic nature of this underdiagnosed disorder, because early detection and dietary treatment may help avoid invasive procedures and reverse liver involvement.
